# Designing and implementing a longitudinal study of children with neurological, genetic or metabolic conditions: *charting the territory*

**DOI:** 10.1186/1471-2431-10-67

**Published:** 2010-09-20

**Authors:** Harold Siden, Rose Steele, Rollin Brant, Susan Cadell, Betty Davies, Lynn Straatman, Kimberley Widger, Gail S Andrews

**Affiliations:** 1 Faculty of Medicine, University of British Columbia, Vancouver, BC, Canada; 2 School of Nursing, York University, Toronto, ON, Canada; 3 Child & Family Research Institute, University of British Columbia, Vancouver, BC, Canada; 4 Faculty of Social Work, Wilfrid Laurier University, Kitchener, ON, Canada; 5 School of Nursing, University of Victoria, Victoria, BC, Canada; 6 Faculty of Nursing, University of Toronto, Toronto, ON, Canada

## Abstract

**Background:**

Children with progressive metabolic, neurological, or chromosomal conditions and their families anticipate an unknown lifespan, endure unstable and often painful symptoms, and cope with erratic emotional and spiritual crises as the condition progresses along an uncertain trajectory towards death. Much is known about the genetics and pathophysiology of these diseases, but very little has been documented about the trajectory of symptoms for children with these conditions or the associated experience of their families. A longitudinal study design will help to close this gap in knowledge.

**Methods/Design:**

*Charting the Territory *is a longitudinal descriptive, correlational study currently underway with children 0-19 years who are diagnosed with progressive neurological, metabolic, or chromosomal conditions and their families. The purpose of the study is to determine and document the clinical progression of the condition and the associated bio-psychosocial-spiritual experiences of the parents and siblings age 7-18 years. Approximately 300 families, both newly diagnosed children and those with established conditions, are being recruited in six Canadian cities. Children and their families are being followed for a minimum of 18 months, depending on when they enroll in the study. Family data collection will continue after the child's death if the child dies during the study period. Data collection includes monthly parental assessment of the child's symptoms; an annual functional assessment of the child; and completion of established instruments every 6 months by parents to assess family functioning, marital satisfaction, health status, anxiety, depression, stress, burden, grief, spirituality, and growth, and by siblings to assess coping and health. Impact of participation on parents is assessed after 1 year and at the end of the study. Chart reviews are conducted at enrollment and at the conclusion of the study or at the time of the child's death.

**Discussion:**

Knowledge developed from this study will provide some of the first-ever detailed descriptions of the clinical symptom trajectory of these non-curable progressive conditions and the bio-psychosocial-spiritual aspects for families, from diagnosis through bereavement. Information about developing and implementing this study may be useful to other researchers who are interested in designing a longitudinal study.

## 

Almost half of non-traumatic deaths in childhood in Canada are from progressive metabolic, neurological, or chromosomal conditions (n = ~1250/year) [1] and financial costs associated with caring for these children are estimated at $1.17 billion annually [2]. Children with these conditions and their families anticipate an unknown lifespan, endure unstable and often painful symptoms, and cope with erratic emotional and spiritual crises as the condition progresses along an uncertain trajectory towards death.

For the majority of children, a cure for these diseases, or a specific treatment that will significantly prolong life, does not exist despite the treatment options that have become increasingly available. Pediatric palliative care, therefore, should begin at diagnosis and focus on anticipating and relieving the child's symptoms while supporting the family to ensure the best possible outcomes in the face of certain death. Unfortunately, there is a lack of research evidence on which to base such care [3-7]. The terminal outcome of the disease may be known, but the onset, timing, patterns, and severity of symptoms are not; consequently, best practices and strategies for symptom relief are also unknown. Furthermore, little is understood about the bio-psychosocial-spiritual impact on these families and how to support them.

There is a critical need to develop a solid understanding of disease progression and the experiences of these children and their families over time. We are currently conducting an innovative 5-year longitudinal study to identify and track children diagnosed with progressive metabolic, neurological, or chromosomal conditions and their families to determine and document the clinical progression of the condition and the associated bio-psychosocial-spiritual experiences of the family. Our goal is to close the key knowledge gaps and provide new information about the best care for these vulnerable children and their families. Our purpose in this paper is to describe the rationale, development, design, and implementation of this study to assist other researchers who may be interested in conducting longitudinal research.

## Background

Our research team, *Transitions in Pediatric Palliative Care and End of Life*, was funded by the Canadian Institutes of Health Research (CIHR) under the New Emerging Team Program (NET) (PET-697#). Led by Dr. Hal Siden and known as PedPalNet, this team was conceived to develop a sustainable research program focused on creating knowledge to optimize provision of care for children with life-threatening conditions. As far as we are aware, this is the first interprofessional, multi-centre team of researchers in pediatric palliative care in the world. We work together to develop specific research projects, obtain project operating funds, attract new research collaborators, and increase research capacity through trainee support and mentoring.

In an early PedPalNet project, we conducted a Delphi process to identify high-value areas for Canadian pediatric palliative care clinicians and researchers. Through that project, we identified four thematic areas, three of which have particular relevance to this current study: (1) identifying what matters most to families; (2) providing relief of symptoms; and (3) developing evidence to support best practice [8]. A foundational cohort study to identify family experiences and symptom courses is a logical sequela to the Delphi project.

Dr. Rose Steele, one of the co-principal investigators for the current study, previously conducted research with families living with a child who has a neuro-degenerative illness. The resulting grounded theory identified the basic social process of 'navigating uncharted territory' and outlined the trajectory, impact on the family, factors that modified the experience, and strategies used by families to manage the experience [9-13]. Lack of information about what could be expected in the illness trajectory was identified as seriously hampering a family's ability to manage the experience. The current study, *Charting the Territory*, builds on the grounded theory study by focusing on children with a wider range of progressive metabolic, neurological, or chromosomal conditions and by using a longitudinal design to describe the symptom trajectory and further understand the whole experience for the child, siblings, and parents from a quantitative perspective.

### Life-Threatening Conditions in Children

Two seminal reports from the United Kingdom [14] and the United States [7] described the broad spectrum of diagnoses of life-threatening conditions encountered in children. These conditions are physiologically diverse, vary in life expectancy and treatments, and more or less fall into four Quadrants [14] (see Table 1).

**Table 1:** Quadrants of Life-Threatening Conditions in Children[14]

Quadrant 1	Quadrant 2
Life-threatening conditions for which curative treatment may be feasible but can fail.	Conditions where premature death is inevitable, but long periods of intensive treatment aimed at prolonging life and allowing participation in normal activities
*(e.g., cancer, irreversible organ failures)*	(e.g., cystic fibrosis)

**Quadrant 3**	**Quadrant 4**

Progressive conditions without curative treatment options, where treatment is exclusively palliative and may extend over many years.	Irreversible but non-progressive conditions with severe disability susceptible to health complications and premature death.
*(e.g., neurodegenerative, metabolic disease)*	*(e.g., anoxic brain injury, severe cerebral palsy)*

Our study focuses on children with Quadrant 3-type conditions. Quadrant 3-type conditions are those without cure or life-prolonging treatment; good symptom management and treatment of comorbid illnesses are emphasized (e.g., aspiration pneumonia) but do not alter the underlying pathophysiological process. The process remains inherent in the disease condition. Examples of diseases that are commonly thought of as fitting into this category include: leukodystrophies, spinocerebellar degenerative diseases, and some chromosomal anomalies, including aneuploid states. Even though the specific diagnoses that tend to fall into Quadrant 3 are varied and numerous, clinically they are similar in functional impairments and diversity of symptoms that limit quality and duration of life.

The number of children with rare, progressive diseases that fall into Quadrant 3 is large relative to the other Quadrants and these children require significant health care attention, yet surprisingly little has been comprehensively explored and documented about the illness trajectory of these conditions. The onset of the disease and symptoms and the length of survival time for these children are virtually unexplored. We chose to conduct research with this group for several reasons:

• These children and their families are major users of health care services for acute, chronic, and end-of-life care [2].

• There is a major impact on families caring for these children [15].

• These children account for about 50% of children receiving palliative care [16-22].

• As a group, Quadrant 3-type conditions are a significant cause of death in childhood [1,19,23,24].

• There is a lack of research in this group compared with other areas, such as cancer [25].

### Knowledge Gaps

Both positive and negative outcomes of the experience have been documented for parents and siblings of these children [9-11,13,26-32] but there is a paucity of research addressing the overall experience of the child and family. It is unclear if outcomes are isolated to specific times, if they are present throughout the experience, or if/how they may change over time. A holistic approach that concurrently examines bio-psychosocial-spiritual dimensions for family members would allow for a more complete picture of the experience.

Given the prevalence of Quadrant 3-type conditions and the associated physical, psychological, and financial costs, it became clear that a longitudinal, prospective study that follows the child and family for as long as possible as they move from diagnosis through death and into bereavement was imperative to document the natural course of the child's condition and the experience of the family. This study was conceived as a way to address this important research area. Results of this study will inform families and clinicians about what they might anticipate during the child's trajectory towards death.

## Methods/Design

### Study Objectives

Our study is a prospective longitudinal study with three objectives:

a) Establish a cohort of children with non-curable, life-threatening conditions.

b) Determine and document the natural history of the symptom progression of these conditions.

c) Determine and document the bio-psychosocial-spiritual trajectory of parents and siblings.

Specific research questions include:

1. What is the clinical illness trajectory, and its impact, for children with non-curable, life-threatening conditions, specifically: pain, breathing or respiratory problems, feeding difficulties, alertness and interaction changes, sleep problems, seizures, constipation, and functional abilities?

2. What is the bio-psychosocial-spiritual trajectory, and its impact, for families when a child has a non-curable, life-threatening condition, specifically outcome variables of: family functioning; marital satisfaction; parent health, anxiety, depression, stress, burden, spirituality, grief, and growth; and sibling health and coping?

3. Do these child and family outcome variables change over time?

4. What relationships exist among the child and family outcome variables and do these relationships change over time?

### Study Design

In this longitudinal research study, we are using quantitative methods with established instruments and chart reviews. Data collection is taking place along two parallel streams: one examining the child's clinical symptoms, and the other examining bio-psychosocial-spiritual aspects of the experience for family members. Under the current funding, children and their families will be followed for a minimum of 18 months, based on when they enter the study. However, we hope to obtain further funding beyond this initial 5-year study and extend this cohort for a longer duration. We also hope to expand data collection in the future to include qualitative interviews that capture other aspects of the experience. We are recruiting children and families with new diagnoses in order to maximize understanding of the condition trajectory, but we also are including those with established diagnoses as some symptoms may only arise later in the condition course. This approach should provide an opportunity to better understand symptoms and parent/sibling experiences at different times along the trajectory. Some children may die during the study period, but the intent is to follow those families through the course of the illness and into bereavement. This descriptive, correlational design will allow for efficient and effective collection of large amounts of data within a naturalistic setting and for investigation of complex relationships that may exist among the clinical and bio-psycho-social variables.

### Eligibility Criteria

Only children with Quadrant 3-type conditions that fall into the group of progressive metabolic, neurological, or chromosomal conditions are eligible for the study. Children who have been provided with potentially curative therapies, such as stem cell transplantation for metabolic conditions, are excluded. The inclusion list was developed from a literature review, palliative care program data (Canuck Place Children's Hospice, Vancouver), and an iterative review process involving pediatric palliative care physicians and specialists in the fields of Neurology, Genetics, and Metabolic Diseases. The resultant list is in accordance with current approaches to pediatric palliative care [14] and work on mortality in childhood Complex Chronic Conditions [23,33].

**Figure 1:**
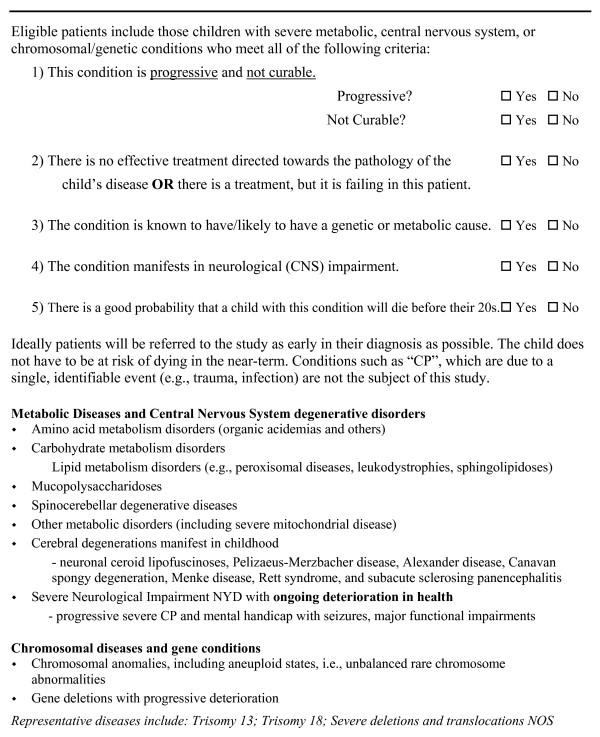
Eligibility Criteria Checklist

Determining who is eligible for recruitment from a theoretical "Quadrant" of conditions initially proved challenging. The Quadrant model, first described as "Categories" by the British charity ACT, is primarily useful as a conceptual framework for understanding the broad variety of children and families receiving pediatric palliative care. Clinically, the model maps broadly onto an identifiable population of eligible children. Before recruitment started, the clinicians began to generate a list of sample eligible diseases in order to establish who would be specifically eligible. From the original list, a system of categories was further developed by geneticists, neurologists, and biochemical disease specialists in our study sites. This list included a category for those conditions that could be treated with curative therapies, but where the child would be eligible if the treatment was failing, and those conditions where the trajectory may not always display the progressive component that is classic for Quadrant 3. The list quickly became iterative in that the clinicians and researchers continue to make notes and compare their new diagnoses to the original list, adding and noting where there is disagreement. This purposive sampling of diagnoses allows the inclusion of children whose disease is progressive, and the constant iterations permit a theoretical model to translate into a practical, useful clinical document to determine eligibility. Potential participants are compared to the list and to an Eligibility Criteria Checklist to determine whether or not to approach the family (see Figure 1).

Families may have various ways of determining who is a member of their family, but for the purposes of this study, "family" includes only the biological, adoptive, step-parents, or legal guardian and biological, adoptive, or step-siblings with whom the affected child lives most of the time. Children in foster care are not eligible because some foster parents only provide short-term care, the foster setting may change during the course of the study, or there may be legal issues with obtaining consent. Families who have multiple children affected with these conditions are eligible, but only those affected children 0-19 years of age are included in the study. A parent may provide data about more than one eligible, affected child. A minimum of one parent must agree to participate on his/her own behalf, plus provide the required information on the ill child throughout the study. In addition, all siblings age 7-18 years are invited to participate. The age ranges were chosen because the instruments we are using have been validated for children of those ages. Participants must speak and write English or French as data collection is only available in the two official Canadian languages.

### Study Recruitment

Three hundred children and their families are being recruited in six cities across Canada (Vancouver, Calgary, Edmonton, Toronto, Ottawa, and Montreal). Sites were chosen to maximize access to the population based on (a) the numbers of children diagnosed annually, (b) the presence of a palliative care team member as an onsite collaborator for recruitment and ethics, and (c) the availability of a specialist clinician/team in Metabolic Diseases, and/or Genetics, and/or Neurology clinics who follow these children and can support access to subjects and clinic records. Prior to commencing recruitment at a participating site, institutional ethics approval was obtained from the Research Ethics Board (REB) of the hospital, as well as from each of the universities where the participating co-investigators held their primary academic positions.

The protocol and all study materials underwent minor modifications to accommodate different requirements at each site. These differences primarily reflect the varying approaches of Research Ethics Boards regarding recruitment. Each REB approved a different method of initial contact between the researchers and the study participants. In provinces where Health Information Protection legislation is strictest, the Research Assistant (RA) is prohibited from contacting families without written approval from at least one of the parents. At other sites, contact information is accessible to the RA who may call or visit with a family in clinic after an introduction from the clinician or clinic nurse. All sites approved the more passive "call back" method, where clinicians mail out invitations from the clinic to the family. Families who are interested call a toll-free 1-800 number for more information. The site RA provides any follow-up information for interested families. None of the sites allow the researchers to recruit the families directly, especially in situations where the Site Investigator also tends to be the child's clinician. This non-coercive procedure is intended to prevent undue pressure on the family to participate.

When the initial recruitment met with challenges, especially where the RA requires a lengthy introduction process or is unable to identify eligible children due to privacy protection laws, two alternate recruitment methods were added to the protocol. In order to maximize the scope of our enrollment, an online recruitment method was proposed. The REB approved clinicians giving out information about our study website. Secondly, disease-specific organizations such as the MPS Society, and other organizations such as the Rare Diseases Foundation and the Canadian Organization for Rare Disorders (CORD), are able to aid in recruitment of eligible families by distributing information about the study on their website and in organizational newsletters. Each organization also has a list of parent support networks to which appeals can be made for assistance with study recruitment.

### Accrual and Attrition

The sample size of 300 is pragmatically motivated as we anticipate that 50 participant families can be recruited in each of the 6 sites. We are confident this sample size is ample for descriptive purposes, e.g., it will enable us to estimate simple proportions with a standard error not exceeding.03. Across the collaborating sites, approximately 300 children are diagnosed annually with the eligible rare, progressive conditions and another 750 children are followed in the clinics. Therefore, about 1650 families would be eligible or become eligible over the 36 month accrual period. Based on previous work investigating recruitment and retention in a longitudinal palliative care study, typically 80-90% of individuals are expected to be receptive to research [34], so a sample size of 300 is feasible. As with any longitudinal study, attrition will likely occur over time, but parents in pediatric palliative care often participate in research as a way of helping others, even when they find it challenging practically (e.g., time) or emotionally (e.g., reliving memories) [11].

We anticipate minimizing attrition through employing a number of strategies highlighted in the literature [35-38]. RAs develop a solid relationship with families through an initial face-to-face visit, telephone contact at prescribed intervals, and letters to thank families for their participation. Continuity of RAs is expected to contribute to the trusting relationship that is needed to help participants feel more connected to and interested in the study over time. Efforts are made to respect families' time and recognize the value of their contribution. RAs schedule data collection at the family's convenience and regularly express verbal and written appreciation for their participation in the study. In addition, along with the blank questionnaire package at each 6-month data collection point, including at baseline, and regardless of how many family members participate, families are given a cheque for $40 as a token of appreciation. We expect that some parents may use the money to help pay for respite while they complete the questionnaires, but there are no restrictions on how parents can spend the token of appreciation.

### Data Collection

Data collection is taking place along two parallel timelines: one recording the child's clinical symptoms monthly and the other examining bio-psychosocial-spiritual aspects of the experience for family members every 6 months. Instruments being used in the study are detailed in Table 2 with information about who completes each instrument and how frequently it is completed. Data are also collected through chart reviews on entry into the study and at the end of the study or at the time of the child's death. Data collection will continue for a minimum of 18 months, depending on how early a family enrols in the study, providing a minimum of 4 data collection points. The method and frequency of data collection were chosen based on longitudinal studies with seriously ill adults that indicate trends/changes should be detectable with this timeline, while minimizing burden as much as possible on individuals who already have significant burdens placed upon them due to the nature of their caregiving experience [37,39]. The questionnaire package and the timing of data collection were previously reviewed and discussed with parents of children with a life-threatening condition who had at least one child enrolled on a hospice program. These parents agreed the study was feasible and the burden on families would not be too great.

**Table 2:** Instruments, Participant Who Completes, and Timing of Data Collection

	Instrument	Who Completes	Timing
**Demographic Information**	Demographic Sheet	Completed by parent	Baseline and updated every 6 months
**Family Level Data**	Family Adaptability and Cohesion Evaluation Scale (FACES III)[61]	Completed separately by each parent who agrees to participate in study	Baseline and every 6 months
**Couple Level Data**	Norton Quality of Marriage Index[62]	Completed separately by each parent who agrees to participate in study, when currently in spousal/common-law relationship	Baseline and every 6 months
**Individual Level Data: Parent**	• SF-12[63] • State-Trait Anxiety Inventory[64] • Center for Epidemiologic Studies Depression Scale (CES-D)[65] • Perceived Stress Scale[66] • Burden Scale[67] • Post-Traumatic Growth Inventory (PTGI)[68] • Spiritual Involvement and Beliefs Scales (SIBS)[69] • Grief Scale • Impact of Participation	Completed separately by each parent who agrees to participate in study	All at baseline and every 6 months, except for Impact of Participation (after 1 year and at end of study)
**Individual Level Data: Affected Child**	• Clinical Symptoms	• Completed by parent	• Baseline and once a month
	• Pediatric Evaluation of Disability Inventory (PEDI^©^)[45-47]	• Completed by RA through observation or parent report	• Baseline and annually
**Individual Level Data: Sibling**	• Kidcope[70]	• Younger version completed by siblings age 7 to 12 years; older version for 13 to 18 years	All at baseline and every 6 months
	• Child Behavior Checklist (CBCL)[71]	• Completed by parent for siblings age 7 to18 years	
	• Youth Self Report (YSR)[72]	• Completed by siblings age 11 to18 years and able to complete by self	

We prefer that baseline data, including written informed consent in accordance with institutional ethics guidelines, are collected in person in order to establish a relationship between the RA and the family from the beginning of the study. However, occasionally a family may be unable to participate in a face-to-face meeting and so we have developed a telephone process for explaining the study and arranging for families to submit written consent and completed questionnaires without actually meeting with the RA. Questionnaire packages are mailed to these families for initial and subsequent data collection. For the newly diagnosed family, baseline data ideally is obtained before changes occur, so within 4-6 weeks of diagnosis, but families where the child has an established diagnosis may be recruited at any stage in the disease process. Assent/consent (the distinction being based on age) is obtained from all child participants who are cognitively able to assent/consent for themselves.

No further consent forms will be signed during the study. Completed questionnaires signify ongoing consent. At each data collection point, families are reminded in the preamble to the questionnaire of their right to refuse participation at any time or to not answer certain questions. To ensure privacy and confidentiality for returns from multiple members of a family, separate envelopes are included for each participating subject to seal their questionnaires before placing them in the larger pre-paid, self-addressed return envelope.

#### Clinical symptoms stream

Data about the ill child's clinical symptoms are collected monthly using an online instrument designed by the research team in consultation with other experienced Canadian clinicians, families, and a psychometrician. The instrument is a modification of the *PediQUEST *symptom recording tool and the revised *Memorial Symptom Assessment Scale (7-12)*, developed for symptoms in children with cancer [40-43]. Symptoms are being tracked for timing of onset (symptom latency) [44], frequency, and distress. Assessed symptoms include difficulties with breathing or respiration, feeding issues, pain, alertness and interaction, sleep issues, seizures, and constipation. Parents of children who received palliative care services from the Canuck Place Children's Hospice reviewed the instrument and affirmed that it includes the most common and/or troublesome symptoms. The instrument takes 6 to 10 minutes to complete and parents may choose either an online or telephone method of completion. If the parent chooses the online method, s/he receives an email every month with a reminder to visit the secure website and complete a web survey. If the parent chooses the telephone method, the RA calls at monthly intervals to administer the instrument and enter the scores manually into the online system while the parent reports on the child's symptoms. Parents were given the choice of administration method for the symptoms' instrument to allow flexibility and accommodate any families lacking Internet access.

With assistance from the RA, the parent assesses the ill child for cognitive and motor ability on enrolment into the study and annually using the Pediatric Evaluation of Disability Inventory (PEDI) [45-47]. In addition to measuring functional abilities, the PEDI evaluates the amount of Caregiver Assistance and Modifications required in caring for the child. Data are collected using this instrument from the time the family enrolls in the study until the time of the child's death or the end of the data collection period, whichever comes first. RAs complete the Caregiver Assistance and Modifications section with the designated parent. To minimize potential error, all RAs involved with this study were trained to complete the PEDI by an occupational therapist. Further, to assess whether there is consistency across the RAs' ratings, inter-rater reliability testing was completed. Analysis of the reliability involved calculating the Intra-class Correlation Coefficients (ICC) for the RA scores on a sample case from the manual. Excellent correlation among the RAs was found for Caregiver Assistance, with an ICC value of 0.959 overall (Self-Care: 0.865; Mobility: 0.976; Social Function: 0.760). Testing on the Modifications scale showed an ICC value of 0.651 overall (Self-Care: 0.550; Mobility: 0.674; Social Function: 0.400). Reliability was high for the Caregiver Assistance scale, and moderate-to-low for the Modification scale. RAs received feedback about ways to improve their administration of the tool in order to maintain ongoing reliability.

Health records are reviewed by the RA at baseline to document the circumstances surrounding the diagnosis and the period leading up to the diagnosis, as well as the prevalence and incidence of symptoms recorded since diagnosis. If the child dies during the course of the study, a chart review is conducted to document details of the care provided just prior to the death and the circumstances of the death. Chart reviews also will be conducted for living children living at the conclusion of the study.

#### Bio-psychosocial-spiritual stream

Data about the bio-psychosocial-spiritual experiences of family members are collected at 6 month intervals, from the time the family enrolls in the study until 6 months prior to the end of the study (to allow for final analyses and write-up), even if the child dies beforehand. The outcomes being measured in the study include: family functioning; marital satisfaction; parent health, anxiety, depression, stress, burden, spirituality, grief, and growth; and sibling health and coping. Most of the measures have demonstrated reliability and validity and have been widely used in previous research with similar populations. The exception is the instrument used to measure grief, which was designed by the research team in consultation with other experienced Canadian researchers. The instrument is a modification of the Inventory of Social Support (ISS), a 5-item self-report questionnaire about what helps or makes it harder to cope with grief [48]. The questionnaire package can be completed in about one hour, as estimated by parents who pilot-tested the instruments.

Unlike the clinical symptom stream where data are collected online or by telephone, the bio-psychosocial-spiritual instruments are completed at home and mailed to the main site. This method of data collection allows family members to complete questionnaires in their own home, at their own pace, rather than having to commit to a potentially lengthy interview time. At baseline, the RA obtains permission to meet with the family during a routine clinic visit or at another more convenient location such as the family home, whichever is more amenable to the family. Once the baseline visit is complete, no further face-to-face meetings with the family are required. If the timing is appropriate and participants want to complete subsequent questionnaires face-to-face with the RA, then they may do so at the clinic visit.

Some of the children will likely die during the course of the study; indeed, we already have faced this outcome with one family. Clinicians involved with the child and family notify the site RA when a child dies. The RA immediately mails a hand-written card of condolence to the family. The RA also conducts a chart review to document the circumstances of the death. Other data collection is suspended for the family until 6 months after the child's death. After that time, the RA contacts the family by telephone to express condolences to the family and obtain verbal agreement for continued participation in the study. Data collection in the bio-psychosocial-spiritual stream then continues at 6 month intervals until the end of the study. Demographics are updated at 6 month intervals throughout the study and into the bereavement period.

### Analysis Plan

Regardless of data collection method (in-person, on-line, telephone, or mail) all data from the multiple sites are entered into Daciforms, a single web-based database (Dacima Software, Inc., Montreal, QC). Daciforms allows researchers at multiple locations to enter and review data online. Each study staff member is assigned a unique password and Site ID that allows secure access to the centralized data. From Daciforms, data are exported into SPSS (IBM, Chicago) for descriptive analysis. Once we have sufficient data points, advanced data analysis and modeling will be performed using the open-source R statistical package (GNU General Public License). Analysis for the clinical symptoms stream involves describing the frequency and severity of symptoms based on the clinical symptom instrument. Following the examination of simple descriptive tables, we will fit longitudinal ordinal regression models including variables representing condition type (neurological, metabolic, or chromosomal), time from diagnosis, age, and sex. We will apply both hierarchical random effect [49] and GEE (generalized estimating equation) [50] models. Akaike's Information Criterion (AIC) [51] will be used to select terms (including interactions) in random-effect models; resulting models will be re-fit using GEE to provide population-level estimates.

The symptom instrument includes items on change relative to previous questionnaire administration based on the expectation that ceiling effects will be modulated by symptom progression. To capture this phenomenon, we are defining a "symptom breakthrough" as occurring if a symptom's severity is at ceiling at time ***t ***but reported to have worsened at time ***t+1***. We will apply longitudinal logistic regression models following the general strategy outlined previously to describe patterns of incidence.

Bio-psychosocial-spiritual measures will be analyzed in a similar manner with descriptive statistics and longitudinal regression models, and we will incorporate random effects to allow for correlation within respondents over time and between respondents within families. Relationships between symptom progression and bio-psychosocial-spiritual measures will be investigated in an exploratory fashion. Due to the complex nature of the data analysis, it was important to involve a statistician (RB) from the outset.

## Ethical Issues

As part of designing the study, and in order to preemptively address potential ethical concerns, we consulted with parents of children who received pediatric palliative care at Canuck Place Children's Hospice in Vancouver. These parents provided advice on timelines and frequency of data collection. Parents highlighted two issues: (1) the crucial need for this type of research and (2) the potential need for emotional support to families. RAs are sensitive to this potential need and have been trained in how to communicate with families and to ascertain whether support is needed. In addition, a detailed list of institutional and community resources to support families is available for each site and is given to families on admission to the study. Families also are reminded at each data collection point and in a semi-annual newsletter that the list of resources is available. The lists of local resources are updated semi-annually and posted on the study website.

## Limitations

One limitation that arose early on was the challenge of defining a theoretical group of children with Quadrant 3 conditions using a rigorous "Inclusion/Exclusion Criteria" checklist. Initially, clinicians were unsure what Quadrant to categorize a diagnosis under and which children could participate. However, the evolving Eligible Disease List that was built from the ground up has involved the clinicians in determining eligibility and has encouraged their participation in refining the eventual criteria. Rather than dictate the eligibility, the list has been strengthened by ongoing discussion and iterative review. While the current study focuses on families of children with Quadrant 3-type life-threatening conditions, it is anticipated that the method may be applied to other groups of diseases. A similar process could be used to identify eligibility in other studies. Researchers in future studies could identify similarities and differences amongst the Quadrants and build a comprehensive picture of important strategies within pediatric palliative care to ensure optimal care for all affected families.

Another potential limitation inherent in the current study design results from using multiple modalities of data collection. We chose to use multiple modalities of data collection (online, phone, written, and in-person) throughout the study in order to provide the most flexibility for participating families. There is some concern in the literature that people respond differently to questions, particularly sensitive ones, based on the method used to answer questions [52]. However, we believe that it is most important for families to have some choice in how they take part in the study and to choose what will fit best with their schedule, lifestyle, and familiarity with technology. This flexibility is important in minimizing family burden related to the study and attrition of families from the research. Our final data analysis will reveal any differences in mode of data collection and its possible impact on the research findings.

Lastly, instruments used in the study design are entirely quantitative. The published instruments demonstrate strong reliability and validity, therefore they were the first choice for initial measurement of this uncharted population. However we recognize the need for and importance of also collecting qualitative data from families. We have already had strong feedback from parents about how important it is to "sit and talk" with the families in order to better understand their full experience. Our hope is to obtain future funding, so that we can expand on quantitative findings of our study through interviews.

## Discussion

*Charting the Territory *is a distinctive study because of the focus on childhood conditions without cure or life-prolonging treatment; use of a prospective, longitudinal design; and simultaneous attention to the affected child, siblings, and parents. There is some reluctance to conduct research with this population because of the perceived negative impact on vulnerable families, however there is mounting evidence to suggest that participation in research during the illness experience and after a child's death is a positive experience for participants and should be conducted. Some studies have highlighted parents' eagerness to share the story of their child's illness and death and to provide input to help other families [53-55]. While parents report that discussions of their child's diagnosis and/or death are at times difficult, the vast majority of parents deny any negative impact or distress caused by participating in research [56-60]. However, very few studies have included a systematic evaluation of the impact of participating in this type of research, and none of the published studies evaluated a longitudinal study design.

To add to the small body of existing research and to guide the study team in development of future longitudinal studies with similar populations, we have incorporated a component to evaluate the impact on parents of participating in the study. This evaluation will be completed by all participating parents through a written survey after one year of enrolment in the study and at the end of the study. As well, at the end of the study the RA will contact the designated parent by telephone to debrief the experience and formally end the relationship with the family. Any suggestions for how the process or procedures of the study could be improved upon will be sought formally at that time, but families also are encouraged to provide feedback throughout the study. If any other family members wish to speak directly with the RA at the end of the study to provide feedback or say goodbye, this conversation will be arranged. A final personalized thank-you and good-bye letter will be sent by the RA on behalf of the research team to the family after this closure call.

We expect that this research will advance knowledge in the fields of family caregiving, subspecialty pediatrics, pediatric palliative care, and bereavement by providing some of the first-ever detailed descriptions of the clinical symptom trajectory and bio-psychosocial-spiritual aspects for these conditions. We will close gaps in knowledge and contribute to the limited, existing body of knowledge specific to Quadrant 3-type conditions in the emerging area of pediatric palliative care. Extending existing research will enable policy makers and practitioners to better understand the realities of families where a child has a progressive, life-threatening condition. Baseline information about critical periods for symptom management and psychosocial support will enable the design of appropriate intervention and support strategies.

Specifically, the study will provide information to families about what they might expect as the child's illness progresses; assist with the development of symptom assessment and evaluation tools, especially in non-verbal children; provide critical information to pediatricians, metabolic, genetics, and neurology specialists, family and child health professionals, and pediatric palliative care clinicians that will assist them to better support children and families; provide a cohort and data to support interventional research in symptom management and care of families; and lay the groundwork for future studies. As noted by the parents, this study will help them learn about expected trajectories; they will then use this knowledge in decision-making and in advocating. Currently, without this knowledge parents and health professionals continue to 'navigate uncharted territory.' In this study the map needed to anticipate outcomes and to guide informed decision-making throughout their journey will be developed.

## Competing interests

The authors declare that they have no competing interests.

## Authors' contributions

All authors made substantive intellectual contributions to conception and design of this study and manuscript. HS and RS were the lead authors responsible for initial drafting of the manuscript. All others, especially KW and GA in the early revision stages, revised it critically for important intellectual content. RB ensured the accuracy of the statistical information. All authors read and approved the final manuscript.

## Authors' information

Dr. Harold Siden, MD, MHSc, FRCPC   Clinical Associate Professor, Faculty of Medicine, Department of Pediatrics, University of British Columbia  Developmental Neurosciences & Child Health, University of British Columbia  4480 Oak Street, Room F612A  Vancouver, BC V6H 3V4 Canada  Tel:  (604) 875-2776  Fax: 604-875-2384  E-mail: hsiden@cw.bc.ca    

Dr. Rose Steele, RN, PhD  Professor, School of Nursing  Room 342 HNES Building   York University  4700 Keele Street  Toronto, ON M3J 1P3 Canada  Tel:  (416) 736-2100 ext. 40556 (work)  Fax:  (416) 736-5714  E-mail: rsteele@yorku.ca    

Dr. Rollin Brant, PhD  Professor, Department of Statistics  University of British Columbia  Child and Family Research Institute  4480 Oak Street, L408  Vancouver, BC, V6H 3V4 Canada   Tel: 604-875-2000 x5389  Tel: 604-875-3570 (main office)  Fax: 604-875-3569  E-mail: rollin@stat.ubc.ca    

Dr. Susan Cadell, PhD, MSW  Associate Professor  Director, Manulife Centre for Healthy Living  Lyle S. Hallman Faculty of Social Work  Wilfrid Laurier University  120 Duke St. West  Kitchener, ON N2H 3W8 Canada  Tel:  (519) 884-0710 Ext. 5252 (office)  Fax: (519) 888-9732  E-mail: scadell@wlu.ca   

Dr. Betty Davies, RN, PhD, FAAN  Professor Emerita, School of Nursing   University of California San Francisco  Professor and Senior Scholar, School of Nursing  University of Victoria  PO Box 1700 STN CSC  Victoria, BC V8W 2Y2 Canada  Tel: (250) 721-6467  Fax: (250) 721-6231  E-mail: betty.davies@nursing.ucsf.edu    

Dr. Lynn Straatman, MD, FRCPC  Clinical Assistant Professor, Faculty of Medicine, Department of Cardiology, University of British Columbia  c/o Canuck Place Children’s Hospice  1690 Matthews Ave.  Vancouver, BC V6J 2T2 Canada  Tel:  (604) 806-9356 (office)  Fax: (604) 806-8763  E-mail: Lynn.Straatman@vch.ca     

Kimberley Widger RN PhD(c) CHPCN(C)  Lawrence S. Bloomberg Faculty of Nursing University of Toronto  155 College Street, Suite 130  Toronto, ON, M5T 1P8 Canada  Tel: 416-946-3928  Fax: 416-978-8222  E-mail: kim.widger@utoronto.ca    

Gail S Andrews, MEd  Developmental Neurosciences & Child Health   University of British Columbia  4480 Oak St., Room F615   Vancouver, BC V6H 3V4 Canada  Tel:  (604) 875-2000 Ext. 5345   Fax: 604-875-2384  E-mail: gandrews@cw.bc.ca  

## Pre-publication history

The pre-publication history for this paper can be accessed here:

http://www.biomedcentral.com/1471-2431/10/67/prepub
